# Relationship between facial morphology, anterior open bite and non-nutritive sucking habits during the primary dentition stage

**DOI:** 10.1590/2176-9451.19.3.108-113.oar

**Published:** 2014

**Authors:** Melissa Proença Nogueira Fialho, Célia Regina Maio Pinzan-Vercelino, Rodrigo Proença Nogueira, Júlio de Araújo Gurgel

**Affiliations:** 1 Professor, School of Dentistry, CEUMA Univeristy, UNICEUMA.; 2 Assistant professor, Department of Orthodontics, School of Dentistry, CEUMA Univeristy, UNICEUMA.; 3 Assistant professor, Brazilian Dental Association/Maranhão.; 4 Assistant professor, Department of Orthodontics, CEUMA University, UNICEUMA.

**Keywords:** Open bite, Face, Primary dentition

## Abstract

**Introduction:**

Non-nutritive sucking habits (NNSHs) can cause occlusal alterations, including anterior open bite (AOB). However, not all patients develop this malocclusion. Therefore, the emergence of AOB does not depend on deleterious habits, only.

**Objective:**

Investigate a potential association between non-nutritive sucking habits (NNSHs), anterior open bite (AOB) and facial morphology (FM).

**Methods:**

176 children in the primary dentition stage were selected. Intra and extraoral clinical examinations were performed and the children's legal guardians were asked to respond to a questionnaire comprising issues related to non-nutritive sucking habits (NNSHs).

**Results:**

A statistically significant relationship was found between non-nutritive sucking habits (NNSHs) and anterior open bite (AOB). However, no association was found between these factors and children's facial morphology (FM).

**Conclusions:**

Non-nutritive sucking habits (NNSHs) during the primary dentition stage play a key role in determining anterior open bite (AOB) malocclusion regardless of patient's morphological facial pattern (FM).

## Abstract (pt)

**Introdução:**

os hábitos de sucção não nutritiva podem causar alterações oclusais, como, por exemplo, a mordida aberta anterior (MAA). No entanto, nem todos os pacientes desenvolvem essa má oclusão. Sendo assim, a instalação da MAA não depende apenas da ocorrência do hábito.

**Objetivo:**

avaliar a possível associação entre hábito de sucção não nutritiva, mordida aberta anterior e morfologia facial.

**Métodos:**

foram selecionadas 176 crianças na fase de dentição decídua completa. Exames clínicos intra- e extrabucais foram realizados, e um questionário, com aspectos relacionados aos hábitos de sucção não nutritiva, foi aplicado aos responsáveis.

**Resultados:**

encontrou-se uma relação estatisticamente significativa entre as variáveis hábito de sucção não nutritiva e MAA. Entretanto, não houve associação desses fatores com a morfologia facial da criança.

**Conclusão:**

a presença dos hábitos de sucção não nutritiva tem associação para a determinação da má oclusão de MAA, independentemente do padrão facial morfológico na dentição decídua.

## INTRODUCTION AND LITERATURE REVIEW

Much has been published about facial morphology (FM) analysis.^1,2,3^ Orthodontic diagnosis on children requires not only proper occlusal and model analyses, but also a thorough assessment of facial configuration for growth prediction. In most cases, growth assessment means advancing a prognosis and, as a consequence, reducing the need for a more complex treatment in future.^4^

Anterior open bite (AOB) is one of the most common malocclusions in the primary dentition, and non-nutritive sucking habits (NNSHs) are among its etiologic factors.^1,5-9^ Continuous thumb and pacifier sucking habits hinder proper teeth and alveolar processes development, especially in the anterior region.^10^

Long-facial morphology (dolichofacial) is closely associated with the development of AOB due to counterclockwise rotation of the mandible and consequent lip incompetence.^10^

AOB is the most prevalent malocclusion in primary dentition.^2,10-13^ It is very often associated with the presence of NNSHs,^6,7,8^ however, it tends to self-correct when the habit is eliminated at an early stage.^14^

To this day, the effect of facial morphology (FM) on growing patients with complete primary dentition and NNSH is seldom found in the orthodontic literature.^15^ Most studies focusing on the association between facial morphology and AOB - with or without NNSH - consider patients in the mixed dentition stage.^11,16,17^

In 2004, Katz et al^1^ examined 4-year-old children in the primary dentition stage to assess the relationship between NNSHs, FM and malocclusions. The authors found no association between facial morphology and malocclusion.

Despite the strong correlation between NSSHs and AOB,^18-22^ not all children develop this malocclusion of which determinant factors are NNSH-related (duration, frequency and intensity) as well as patient-related (alveolar resistance and growth pattern). In light of a notorious lack of studies focusing on patients in the primary dentition stage, the aim of this study was to test the null hypothesis that there is no relationship between facial morphology (FM), anterior open bite (AOB), and non-nutritive sucking habits (NNSH).

## MATERIAL AND METHODS

This cross-sectional study was conducted with subjects randomly selected from a population of children attending elementary schools in the city of São Luís, Brazil. The sample comprised 176 children with primary dentition (96 females and 80 males) aged between 3 and 6 years old (mean age: 4.9 years, maximum age, 6.8 years and minimum age, 2.1 years). The sample was divided into two groups: Group 1 consisting of children with NNSHs, and group 2 comprising children without any history of NNSHs.

The following inclusion criteria were applied: Children in the primary dentition stage; presence of all primary teeth; absence of extensive caries; no significant anomalies of tooth shape or size; no history of previous orthodontic treatment; NNSH and non-NNSH patients. Additionally, children's legal guardians were required to sign an informed consent form. The exclusion criterion was the absence of the habit, only. This study was approved by the Institutional Review Board of CEUMA University under protocol nº 00785/10.

The study was conducted in three major phases. At first, school principals were presented with the permits issued by the City Health Department, the children were selected according to the inclusion criteria and a second meeting was scheduled with the guardians. Secondly, the children's legal guardians were provided with detailed information about the study, its purpose and how the children would be examined. Thereafter, an informed consent form was signed by those who agreed with the research. NNSH data were collected by means of a questionnaire answered by the children's legal guardians. The questionnaire was applied individually by the researcher herself. Thirdly, the study was geared towards examining the children both intra and extraorally by means of an individual clinical assessment. Intraoral examination was performed under natural light with the child positioned in front of the examiner with their dental arches positioned in centric relation. The examiner was previously calibrated and during clinical examination, had no access to the questionnaire that provided information about the children's sucking habits (blinded).

A flexible ruler was used to assess overbite. Measurements were taken from the incisal edge of the maxillary right central incisor to the incisal edge of the lower right central incisor. Children with negative overbite and linear measurement greater than 1 mm were deemed to have anterior open bite (AOB).

Extraoral examination was conducted to determine the children's facial morphology (short face, balanced face or long face). The Facial Morphologic^23^ index (FMI) was applied based on the ratio between morphological facial height (MFH) and bizygomatic distance (BD) established by means of the following formula: FMI = MFH/BD. Morphological facial height was determined based on the linear distance between the nasion and gnathion, and the bizygomatic distance between the zygomatic points (Fig 1).

A digital caliper (Mitutoyo Digimatic Caliper 200mm/.0005 "- 8"; cat. No. 500 - 147B; Battery: SR44, serial number: BH012006 - Suzano - São Paulo - Brazil) was used for extraoral measurements. Based on the values obtained, children's facial morphology was then classified into short face (< 83.9), balanced face (84.0 to 87.9) and long face (> 88.0).^1,23^

To ensure reliability, 36 children were randomly selected and re-assessed after 4 weeks. Numeric variables (bizygomatic distance, morphological facial height and overbite) were remeasured and the difference between the first and second measurements was determined. Dependent Student's t test was applied to assess the significance of differences observed between the two measurements, thereby revealing systematic error, according to Houston.^24^ To assess random error, Dahlberg's formula^25^ was employed (Se^2^=Σd^2^/2n).

Qualitative variables were described by absolute (n) and relative (%) frequencies. The *overbite* variable was described by mean and standard deviation.

To investigate the association between sucking habit and variable AOB, sucking habit and facial morphology, as well as the association between FM, AOB and NNSH, chi-square test was applied. Additionally, when the smallest expected frequency was less than or equal to 5, Fisher's Exact Test was applied.

Relationships were also expressed by odds ratio with confidence interval set at 95%.

One-way analysis of variance (ANOVA) was applied to compare AOB severity between the different facial types.

Level of significance was set at 5% (p < 0.05) for all tests. Statistical analyses were performed with Statistica version 5.1 software (StatSoft Inc., Tulsa, USA).

## RESULTS

The error of the method results revealed reproducibility of measurements, since there were no systematic or random errors (Table 1).

**Table 1:** Error of the method - results of paired t test and Dahlberg's formula. ^25^

Occlusal Indexes	1^st^ Measurement	2^nd^ Measurement	*p*	Dahlberg
	Mean ± SD	Mean ± SD		
Bizygomatic distance	100.17 ± 5.08	100.11 ± 5.14	0.10	0.13
Morphological facial height	84.32 ± 3.88	84.30 ± 3.87	0.26	0.08
Overbite	0.58 ± 2.65	0.57 ± 2.64	0.25	0.04

*Statistically significant: p < 0.05

A statistically significant association was found between NNSHs and the presence of AOB (Table 2). Nevertheless, there was no association between FM and AOB (Table 3).

**Table 2:** Association between non-nutritive sucking habits and anterior open bite.

Groups	No AOB		AOB		Odds ratio (IC 95%)	
	n	%	n	%		
NNSH	16	40.0	24	60.0	40	Reference 66.50 (17.99 - 245.80)
No NNSH	133	97.8	3	2.2	136	
Total	149	84.7	27	15.3	176	
c2 = 79.49; p < 0.00*						

* Statistically significant: p < 0.05

**Table 3:** Association between facial morphology and anterior open bite.

Morphology	No AOB		AOB		Total	*Odds ratio* (IC 95%)
	n	%	n	%		
Short face	56	84.8	10	15.2	66	Reference
Balanced face	46	83.6	9	16.4	55	0.91 (0.34 - 2.44)
Long Face	47	85.4	8	14.6	55	0.87 (0.31 - 2.45)
Total	149	84.7	27	15.3	176	
c2 = 0.07; p = 0.96						

*Statistically significant: p< 0.05

Overall, children's FM showed no significant association with AOB malocclusion even in the presence of NNSHs. Children with balanced face and with NNSHs had the greatest percentages of AOB, followed by short-faced children (Table 4).

**Table 4:** Association between NNSHs, AOB and facial morphology.

Deleterious habit and facial morphology	No AOB		AOB		Total	*Odds ratio* (IC 95%)
	n	%	n	%		
NNSH and short face	6	37.5	10	62.5	16	Reference
NNSH and balanced face	5	35.7	9	64.3	14	0.93 (0.21 - 4.11)
NNSH and long face	5	50.0	5	50.0	10	0.56 (0.11 - 2.90)
Total	16	40.0	24	60.0	40	
c2 = 0.57; p = 0.75						

*Statistically significant: p< 0.05

Among children with AOB, those with a short face had more severe anterior open bite, followed by those with a balanced face, and finally, a short face. No statistically significant difference was found between facial morphology and AOB severity (Table 5).

**Table 5:** Comparison among the three facial morphology patterns in terms of overbite in children with AOB, expressed in mm.

Negative overbite			
Facial morphology	Mean ± SD		*p*
Balanced face	-3.67 ± 2.00		0.33
Short face	-4.80 ± 1.75		
Long Face	-3.63 ± 2.00		
ANOVA: F = 1.15			

*Statistically significant: p< 0.05

Finally, test power was applied, since no sample size calculation was performed at baseline to validate the results. Test power results showed that the study sample (n = 176) had 80% power to detect a difference of 22 percentage points in the association test between two variables.

## DISCUSSION

This study was conducted with a sample of children in the complete primary dentition stage, given that AOB malocclusions often occur during this period. Moreover, only a few studies have focused on this phase of occlusion development, since most researches associating habits with malocclusions focus on the mixed dentition stage.^11,16,17^ In addition, studies about facial morphology are scarce both in the mixed dentition and primary dentition stages,^4^ particularly those seeking to correlate facial morphology with the presence of non-nutritive sucking habits and anterior open bite.^1^

In Dentistry and other healthcare areas, there is an ongoing concern about the radiation doses applied to patients. Recent studies have been conducted to assess the acceptable doses employed in the different imaging methods^26^ with the purpose of reducing radiation in humans. Great emphasis has been given to facial analysis. Morphological facial index is a method used to classify facial patterns (facial morphology) without the need to expose the child to unnecessary radiation. For this reason, it was employed in the present study. It should be added that other studies^1,2^ with similar methods were also conducted in the primary dentition stage and previously published in the literature using morphological facial index.

Results yielded with children with non-nutritive sucking habits reveal a 60% occurrence of AOB, which corroborates the values obtained by Sousa et al^2^ (63.4%), but are nowhere near the values found by Katz et al^1^ (35.5%). Other authors also found a positive relationship for the NNSH / AOB ratio.^6-8,19-21^

Results revealed no association between facial morphology, NNSHs and AOB. It is speculated that, although NNSHs act as etiological factors of malocclusions,^5-8^ facial morphology does not interfere in this process as a facilitating factor in the emergence of this malocclusion during primary dentition.^1^

Katz et al^1^ reported that the different types of facial morphology and NNSHs produce independent effects on malocclusions. Among these is AOB which should therefore be studied separately. These authors^1^ endorse the idea that genetic factors seem to play a less important role than commonly believed, and that many types of malocclusions are actually acquired rather than inherited. However, Cozza et al^11^ refute this idea, stating that chronic non-nutritive sucking habits and the characteristics of facial hyperdivergence (long face) pose significant risks for the development of AOB when occurring together. It should be emphasized, however, that in the aforesaid study^11^ assessments were performed based on the cephalograms of children with a mean age of 9 years and 3 months, and in the mixed dentition stage, which differs from the sample used in the present study.

In this study, it was observed that among all children presenting with AOB, 14.6% had long face pattern, 15.2% short face, and 16.4% balanced face. Thus, the fact that patients with long face exhibited the lowest values in determining the onset of AOB shows an independent relationship between the presence of AOB and long face morphology, as well as other morphologies, in agreement with the results of Katz et al^1^ and Sousa et al,^2^ who applied a methodology that was similar to the one used in this study.

None of the types of facial morphology was prevalent, given that the values of 31.25%, 37.5% and 31.25% found for the balanced face, short face and long face, respectively, showed no statistically significant differences. This contrasts with the findings of Sousa et al^2^ and Silva Filho et al^15^ who argue that balanced face is the predominant pattern. It is believed that this difference in outcomes may be related to the fact that their investigation was carried out in different regions and in populations with different morphological characteristics.

With regard to the relationship between AOB severity and facial morphology, short-faced children exhibited the highest values of negative overbite, followed by balanced-faced, and long-faced children (Table 5). NNSH duration is of paramount importance in determining AOB emergence.^19^ The literature shows that factors such as NNSH duration, frequency and intensity affect AOB severity.^17^ This finding probably explains the higher values of negative overbite observed in short-faced children.

## CONCLUSIONS

According to the methods applied in this study and after careful analysis of results, it seems reasonable to conclude that non-nutritive sucking habits (NNSHs) during the primary dentition stage play a key role in determining anterior open bite (AOB) malocclusion regardless of morphological facial pattern.
